# Localised and unresectable neuroblastoma in infants: excellent outcome with low-dose primary chemotherapy

**DOI:** 10.1038/sj.bjc.6601259

**Published:** 2003-10-28

**Authors:** H Rubie, C Coze, D Plantaz, C Munzer, A S Defachelles, C Bergeron, C Thomas, P Chastagner, D Valteau-Couanet, J Michon, V Mosseri, O Hartmann, on behalf of the Neuroblastoma Study Group of the Société Française d'Oncologie Pédiatrique (SFOP)

**Affiliations:** 1 Unité d'Hémato-Oncologie, Hôpital des Enfants, 330 avenue de Grande Bretagne BP 3119, 31026 Toulouse Cedex 3, France; 2 Service d'Oncologie Pédiatrique, Hôpital de la Timone, Marseille, France; 3 Unité d'Hémato-Oncologie, Hôpital de la Tronche, Grenoble, France; 4 Service d'Oncologie Pédiatrique, Centre OscarLambret, Lille, France; 5 Département de Pédiatrie, Centre Léon Bérard, Lyon, France; 6 Unité d'Hémato-Oncologie Pédiatrique, Hotel Dieu, Nantes, France; 7 Service d'Immuno-Hémato-Oncologie, Hôpital d'Enfants, Nancy, France; 8 Département de Pédiatrie, Institut Gustave Roussy, Villejuif, France; 9 Département d'Oncologie Pédiatrique, Institut Curie, Paris, France

**Keywords:** infants, neuroblastoma, unresectable, chemotherapy

## Abstract

The purpose of this study was to evaluate the efficacy of low-dose chemotherapy in infants with localised and unresectable neuroblastoma (NB). All consecutive infants with localised NB and no N-*myc* amplification were eligible in the SFOP-NBL 94 study. Primary tumour was deemed as unresectable according to imaging data showing any risk of immediate resection. Diagnostic procedures and staging were conducted according to INSS recommendations. For children, provided that they had no threatening symptom (i.e. vital risk or dumb-bell NB with neurologic deficit), chemotherapy consisted in low-dose cyclophosphamide (5 mg^−1^kg day^−1^ × 5 days) and vincristine (0.05 mg kg^−1^ at day 1)–CV and repeated one to three times every 2 weeks until surgical excision can be safely performed. No postoperative treatment was given. Between January 1995 and December 1999, 134 consecutive infants with localised NB were registered in the study, of whom 39 had an unresectable NB without N-*myc* amplification. Among them 28 had no threatening symptom and received CV according to the protocol. Objective response was observed in 14 (50%) and the other 14 were given second-line chemotherapy because of no response. Surgery was attempted in 38 patients including 14 after CV alone, leading to complete resection in 23. Relapses occurred in four patients all local. Survival and event-free survival were 100 and 90±5% with a median follow-up of 55 months (range 33–93). In conclusion primary low-dose chemotherapy without anthracyclines is efficient in about half of the infants presenting with an unresectable NB and no N-*myc* amplification, allowing excellent survival rates without jeopardising their long-term outcome even for nonresponding patients who received standard regimen.

Neuroblastoma (NB) is the most common solid tumour of early childhood and accounts for the main cause of cancer in infants (Bernstein *et al*, 1992). Indeed, nearly one-third of NBs are diagnosed in children younger than 1 year. Half of the patients present with localised disease (Hartmann *et al*, 1983; Rosen *et al*, 1984) and gross surgical excision is considered as the main requirement for cure (Evans *et al*, 1976; Le Tourneau *et al*, 1985). Primary surgery can be performed in about 50% of these children and reported survival rates are high (De Bernardi *et al*, 1995). Conversely, unresectable tumours usually have a poorer outcome, unless secondary radical excision can be performed (Haase *et al*, 1989, Tsuchida *et al*, 1989). Consequently, the efficacy of primary chemotherapy in allowing subsequent resection is of outstanding importance (Garaventa *et al*, 1993; West *et al*, 1993). Recently, we reported improved outcome in such patients after primary chemotherapy including carboplatin and etoposide with a 90% survival rates and children with unresectable NB and no N-*myc* amplification fared as well as those undergoing primary surgery (Rubie *et al*, 1998). Moreover, such a strategy resulted in survival rates over 95% in infants (Rubie *et al*, 2001). However, immediate haematological toxicity and transfusion-related risks and possible long-term side effects of such treatment administered in young children may limit its use, although preliminary results of their follow-up showed neither renal nor audition deterioration (Bergeron *et al*, 2000). Furthermore, this regimen included anthracyclines and we are concerned about the possible long-term consequences on cardiac function. Those considerations prompted us to evaluate the efficacy of a presumably less toxic chemotherapy with low-dose cyclophosphamide and vincristine (CV) in order to attempt a safe surgical excision and ultimately decrease the risks of long-term sequelae (NBL 94 study).

## PATIENTS AND METHODS

### Patient population

All consecutive and untreated children younger than 12 months at diagnosis, and referred to SFOP institutions, were eligible in the study. Evaluation at diagnosis included CT scan or MRI. MIBG and extensive bone marrow staging (at least two evaluable aspirates and two trephine biopsies if possible). Tumour volume was calculated with the product of the theee largest diameters (height, width and thickness). Urinary catecholamines, serum NSE, ferritin and LDH were also measured. Histology of the primary (with either surgical biopsy or tru-cut biopsy) was mandatory to allow both NB diagnosis according to INSS recommendations (Brodeur *et al*, 1993) and MYCN analysis. Only patients with localised NB and no N-*myc* amplification (less than 10 copies per haploid genome) were eligible in the study.

### Surgery

Participating institutions were provided with guidelines for surgical procedures. Resectability was defined according to imaging data. Procedures that would have resulted in the removal of major organs were not recommended unless primary chemotherapy had been administered before any attempt of excision. Tumours defined as unresectable were lesions that crossed and infiltrated the mid-line structures, usually encasing large vessels, and tumours that because of size structure or location, were deemed as difficult to resect without a high risk of rupture of the tumour or of major surgical complications. All children presenting with life-threatening symptoms (cardiac failure, acute respiratory distress, etc) or dumb-bell tumours and neurological symptoms were urgently assigned to more intensive chemotherapy using a combination of carboplatin and etoposide. The appropriateness of treatment allocation was systematically reviewed. Postoperative imaging (CT scan or MRI) was required in all patients 1 month after resection. MIBG scan was recommended in case of macroscopic residue. Postsurgical staging was defined on the basis of surgical, pathological reports and postoperative imaging data.

### Chemotherapy

**Figure 1:**
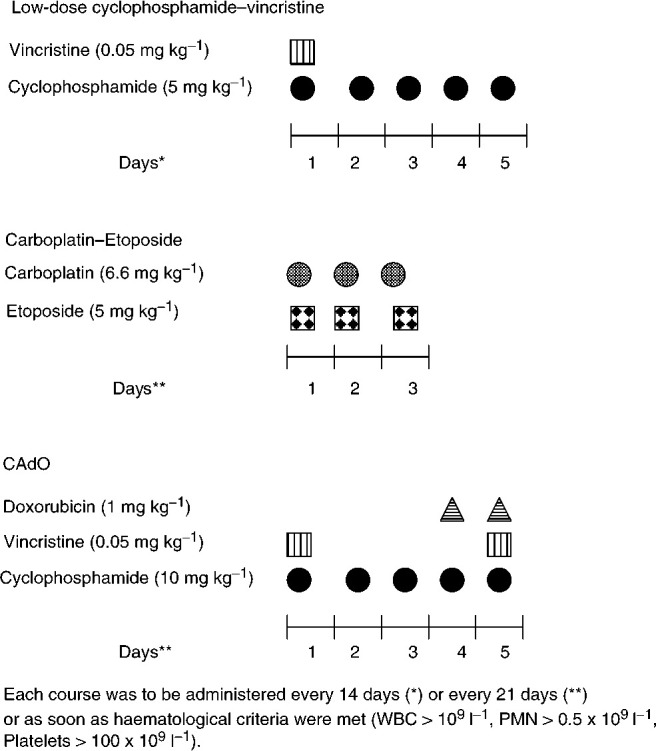
Chemotherapy regimen.

First-line chemotherapy consisted of two courses of low-dose cyclophosphamide (5 mg kg^−1^ for days 1–5) and vincristine (0.05 mg kg^−1^ on day 1) (CV) given at a 2-week interval (Figure 1

). Following a first evaluation after those two courses, two more CV courses were to be administered if tumour shrinkage was over 25% and if the tumour was still unresectable. Resectability was then assessed and surgery was attempted in the absence of risk. In case of either no tumour response after the two first courses of CV, or persisting preoperative risk after four courses, or life-threatening symptoms, or symptomatic dumb-bell at diagnosis, a combination of carboplatin (6.6 mg kg^−1^ on days 1–3) and etoposide (5 mg kg^−1^ on days 1–3) (CE) was given, followed if necessary by two courses of vincristine (0.05 mg kg^−1^ days 1 and 5), cyclophosphamide (10 mg kg^−1^ on days 1–5) and doxorubicin (1 mg kg^−1^ on days 4 and 5) (CAdO) as previously described (Rubie *et al*, 1998). Drug doses were always reduced by 30% in babies under 1 month of age. No postoperative treatment was recommended whatever the quality of surgical excision.

### Evaluation of response to therapy

Response to therapy was assessed according to INRC criteria (Brodeur *et al*, 1993). The value of tumour response, based on reduction of volume that was considered more significant than that of urinary catecholamine excretion, was evaluated during induction therapy (every two courses) before and 1 month after surgery, and then at least every 3 months. As those children were young, response was considered as significant if the tumour shrinkage was over 25% of the volume at diagnosis (objective response–OR) instead of the usual shrinkage of 50% (partial response–PR). Indeed, such an objective effect was considered as encouraging to continue the treatment.

### Stastistical analysis

To prevent selection bias, all consecutive patients with newly diagnosed localised NB in the participating centres were included in the analysis, whatever the treatment actually administered. The probabilities of survival were calculated from the time of diagnosis to death or last follow-up according to the Kaplan–Meier product-limit method (Fleiss, 1981). In the event-free survival (EFS), disease progression or relapse and death, whatever the reasons, were considered as events. Comparisons between mean doses of chemotherapy and proportions were performed with Student's *t*- and *χ*^2^-tests corrected for heterogeneity or Fisher's exact test, respectively (Peto *et al*, 1977). The analysis of EFS times was performed by Cox's proportional hazards models and differences between curves were tested for statistical difference by the log-rank test (Cox, 1977). The multivariate model was not used as no prognostic factor was found. All tests were two-tailed.

## RESULTS

From January 1995 to December 1999, a total of 301 consecutive children with confirmed localised NB were registered in the study. Among them, 134 were infants of whom 82 underwent primary surgical excision. Among the 52 remaining infants presenting with and unresectable primary, N-*myc* was not evaluable in 13 and not amplified in the other 39. Therefore, the present analysis (NBL 94 study) deals with 39 infants with localised unresectable tumour and no N-*myc* amplification, and reports their outcome as of October 2002, 33 months after the last patient's inclusion.

## PATIENT CHARACTERISTICS (TABLE 1)

**Table 1:** Patient characteristics in comparison with the previous cohort (Rubie *et al*, 2001)

	**NBL 90 study**		**NBL 94 study**		
	**Number**	**%**	**Number**	**%**	***P***
Registered	338		385		
Eligible	316/338	93.5	301/385	78.2	<0.001
Infants	152/316	48.1	134/301	44.5	NS
Unresectable	52/152	34.2	52/134	38.8	NS
Evaluable N-*myc*	37/52	71.2	39/52	75.0	NS
No n-*myc* amplification	35/37	94.6	39/39	100	NS
Median age (months, range)	5.4 (1–11)		5.3 (0–11)		NS
Dumb-bell tumour	14	40	15	38	NS

NS=not significant

Most primary tumours were abdominal (64%), INSS stage 3 (97%) and neuroblastoma (97%). As compared to the previous cohort (NBL 90 study), patient characteristics were similar regarding age, gender, location of the primary, stage, histology and N-*myc* status (Table 1). The only difference was patient's eligibility. Actually, as compared to the NBL 94 study, histology was not mandatory at diagnosis in the NBL 90 study, explaining that more children could be included in the former one.

### Primary chemotherapy (Table 2)

**Table 2:** Response to primary chemotherapy and outcome for all patients (*n*=39)

**First-line chemotherapy**	**Response**	**Second-line**	**chemotherapy**	**Overall response**	**Postoperative status**	**Relapse**	**Outcome**
CV, *n*=28	14 OR	CV, *n*=11	None	11 PR	7 CR, 4 VGPR		11 CR
		CE, *n*=3^a^	None, n=2	2 PR	2 VGPR	2 local	2 CR
			CAdO, *n*=1	1 PR	1 VGPR		1 VGPR
	10 NR	CE, *n*=10	None, *n*=6	3 PR	2 CR, 1 VGPR		3 CR
				3 NR	2 CR, 1 VGPR	1 local	3 CR
			CAdO, *n*=4	2 PR	1 CR, 1 PR		2 CR
				2 NR	2 CR		1 VGPR, 1 PR
	4 NE	CV, *n*=3	None	2 PR, 1 NR	3 CR		3 CR
		CE, *n*=1	None	1 PR	1 CR		1 CR

CE, *n*=10	6 OR	CAdO, *n*=4		4 PR	1 CR, 3 VGPR		4 VGPR
		CV, *n*=2		2 PR	1 CR, 1 VGPR		2 CR
	3 NR	CAdO, *n*=2	None	1 PR	1 CR		1 CR
				1 NR	1 VGPR	1 local	1 VGPR
		CE, *n*=1	CAdO, *n*=1	1 PR	1 CR		1 VGPR
	1 NE	CAdO, *n*=1		1 PR	1 CR		1 CR

Observation, *n*=1							1 CR

CV=cyclophosphamide–vincristine; CE=carboplatin–etoposide; CAdo=cyclophosphamide–doxorubicin–vincristine; CR=complete remission; VGPR=very good partial remission; PR=partial remission; OR=objective response; NR=no response.

a Those three children received CE because persisting neurological symptoms or no intraspinal component shrinkage after CV courses (see text).

A total of 29 children were to be given low doses of CV according to the protocol. Among them, one patient presented with an antenatal diagnosis and spontaneous regression of the tumour was observed and neither chemotherapy nor surgery was realised. Thus, 28 received two courses of CV: OR was observed in 14, which led to two additional courses to 11 of them before surgery. The remaining three children presenting with a moderately symptomatic dumb-bell tumour at diagnosis had no significant improvement or response of the intraspinal component after the two first CV courses and received subsequently the CE regimen. Four additional patients were not evaluated correctly after the two CV courses, three of whom were given two additional courses. No significant response was observed in 10 and all received CE according to the protocol. Among the 10 remaining children, six were given CE as first-line chemotherapy either because of symptomatic dumb-bell tumour (*n*=4) or life-threatening symptoms (*n*=2), according to the protocol and four for unknown reason. No relationship could be established between children having threatening symptoms and tumours with other risk factors (i.e. size, LDH, etc). Symptomatic dumb-bell NB was observed in eight children with moderate to severe neurological impairment. Among them, seven received CE according to the protocol, either at diagnosis (*n*=4) or after CV courses because of persisting symptoms or no significant response of the intraspinal component (*n*=3). According to the protocol recommendations, neurosurgical procedure was considered as useless (due to the duration of deficit) or could be avoided in all but one and neurological recovery was observed in five of them. As a whole, the observed response rate (RR) according to first-line chemotherapy was 61% (*n*=20 out of 33 evaluable) and 58% in patients receiving only CV (*n*=14 out of 24). Furthermore, RR to first- and second-line chemotherapy planned by the protocol was 82% (31 out of 38). No significant toxicity, particularly haematological, was observed after CV and the time elapsing between two courses was always less than 2 weeks. The toxicity of CE±CAdO was mainly haematological and easily manageable as already reported (Rubie *et al*, 1998,^2001^). In summary, 14 children received only CV (four courses), 11 were given CV and CE (two courses each), five had CV–CE and CAdO (courses each) and eight received CE and CAdo (two courses each). The cumulative doses of potentially harmful drugs (i.e. doxorubicin–etoposide and carboplatin) was significantly lower in the present protocol than that of the previous regimen given in the NBL 90 study particularly for doxorubicin (2.31 *vs* 4.29 mg kg^−1^, *P*<0.0005) and carboplatin (31.98 *vs* 41.3 mg kg^−1^, *P*<0.05).

### Surgery

Surgical excision was attempted in all children but one (spontaneous regression of the tumour with continuous complete remission 35 months after diagnosis). Among the 38 remaining patients, the result of the procedure was complete resection (CR) in 23, either with a microscopic residue (*n*=18) or without (*n*=5), very good partial remission (VGPR) in 14 and partial remission (simple biopsy) in one. Among children receiving CV only, the results of the procedure was CR in 10 and VGPR in four. There was no severe complication related to surgery. Postoperative treatment was given to three children having residual disease despite protocol recommendations.

### Outcome

**Figure 2:**
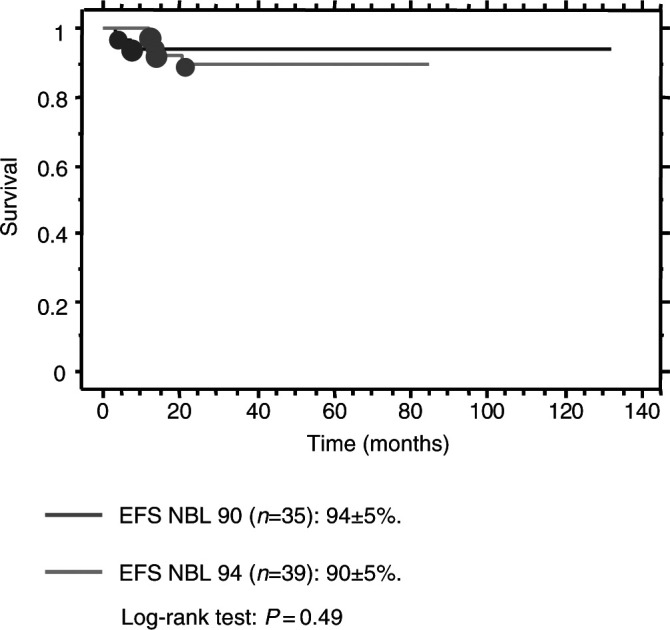
Event-free survival according to the treatment.

As shown in Figure 2

, the overall survival (OS) and EFS were 100 and 90±5% respectively, with a median follow-up of 55 months (33–93). No treatment-related death was observed. Relapse occurred in four children, all local at a median time of 14 months after diagnosis (12–21 months). Salvage therapy included conventional doses of chemotherapy (CE, CAdO, etoposide–cisplatin) followed by surgical excision leading to second CR (*n*=3) or PR (*n*=1) with a median follow-up of 24 months after relapse (15–36).

### Survival and prognostic factors

OS is 100%, and as shown in Figure 2, the EFS rates were similar to those observed in the previous NBL 90 study (90 *vs* 94%, log rank=0.49). No prognostic factor could be identified in that selected population because of the small size of the cohort and the very low number of events.

## DISCUSSION

The outcome of children with localised NB is favourable, particularly in those with resectable tumours (Evans *et al*, 1976; De Bernardi *et al*, 1995). Conversely, unresectable NB often carries a poor prognosis as achieving EFS greater than 50% has proven difficult (Garaventa *et al*, 1993, West *et al*, 1993). In that subset of patients, surgical excision is recognised as being a major step for cure (Le Tourneau *et al*, 1985; Haase *et al*, 1989; Tsuchida *et al*, 1989). Consequently, primary chemotherapy allowing resection is of outstanding importance. The outcome of infants with localised but unresectable tumours is rarely reported and represents a challenging issue to limit the risk of immediate morbidity and treatment-related sequelae. Indeed, the poor prognosis usually associated with unresectable primaries should be balanced with the favourable outcome reported in infants (Evans *et al*, 1976; Hartmann *et al*, 1983).

We reported recently in the NBL 90 study a survival rate over 95% in infants presenting with localised and unresectable NB and with no N-*myc* amplification, but chemotherapy was intensive (Rubie *et al*, 2001). These excellent results may have been due first to a strict selection of patients having a favourable type of NB (i.e. extensive staging including MIBG to eliminate metastatic disease). Second, it has been demonstrated that N-*myc* amplification had a major prognostic value in localised NB, including in infants; indeed, although rare in that subset of patients, N-*myc* amplification has a strong negative influence on outcome (Rubie *et al*, 1997). Last, primary chemotherapy using a combination of two courses of CE followed by two courses of CAdO proved to be very efficient as all infants could undergo safe and satisfying surgical excision as shown in our previous NBL 90 study. However, haematological toxicity was significant, although always manageable, carrying transfusion-related risks. Moreover, although subsequent follow-up showed neither auditive nor renal toxicity (Bergeron *et al*, 2000), the risk of long-term sequelae due to that chemotherapy administered in young children cannot be definitely ruled out. This is the reason why the efficacy of a low-dose CV regimen as a first-line therapy was investigated in that subset of patients, providing no urgent situation (i.e. life-threatening sign or symptomatic dumb-bell tumour) as that regimen is thought to have a lower or delayed efficacy as compared to CE combination. The stratification of treatment could not be performed on other risk factors such as tumour size or location or LDH, since small primaries may cause threatening symptoms. Thus, treatment allocation was based on clinical tolerance of the tumour. Indeed, 20% of patients presented with a symptomatic dumb-bell tumour. In such a situation, any treatment has to be delivered in emergency to avoid neurologic sequelae and we already reported the efficacy of that chemotherapy as an alternative to decompressive laminectomy in order to prevent orthopaedic sequelae as well (Plantaz *et al*, 1996). This strategy also seems appropriate for children presenting with slight neurological symptoms. Indeed, in this study three of four children were given CE as second-line chemotherapy, suggesting that CV is not to be recommended in that situation. Although the number of patients is small, we confirm the relevance of such an approach in this series. As far as other patients are concerned, this strategy proved to be efficient as a safe resection could be attempted in nearly 50% of them after two to four courses of CV, resulting in a continuous remission. Last, EFS of the present cohort is 90%, which is similar to that of the previous series treated with a more intensive primary chemotherapy (Rubie *et al*, 2001).

Thus, it appears that we could obtain the same results while sparing intensive chemotherapy in 50% of these children. Is it possible to go further in the de-escalation process? The usefulness of neoadjuvant treatment may be questioned in such a subgroup of patients. A recent report suggests that apart from surgical excision adjuvant treatment is not necessary, providing no N-*myc* amplification has been found (Kushner *et al*, 1996). However, this is a single-centre study that included only four patients with true INSS stage 3 disease and no dumb-bell tumour, and such results may be difficult to achieve in a multicentre setting. Furthermore, surgery may be also harmful, especially in multicentric studies. We already reported a 3% death rate after postoperative complications among 186 patients who underwent primary surgical excision (Rubie *et al*, 1997). As spontaneous regression of the disease may be observed in very young children, some investigators are evaluating the feasibility of observation only in infants presenting with localised but unresectable NB. Recently, the German group reported spontaneous regression of the primary after biopsy or partial resection in infants with unresectable NB, but the cohort was small and the follow-up still too short to draw any definitive conclusions (Hero *et al*, 2002). Those studies are still underway and their results, providing they are confirmed, may be considered to establish future strategies in multicentric prospective studies.

In conclusion, low-dose chemotherapy is efficient in about half of infants presenting with unresectable NB and no N-*myc* amplification, allowing safe surgical resection and preventing long-term late side effects. Those patients have to be strictly selected (absence of threatening symptom) and followed cautiously in order to benefit from such a de-escalation strategy without jeopardising the excellent outcome. This approach is presently awaiting confirmation in a larger European study.

## References

[bib1] Bernstein ML, Leclerc JM, Bunin G, Brisson L, Robison L, Shuster L, Byme T, Gregory D, Hill G, Dougherty G, Scriver C, Lemieux B, Tuchman M, Woods WG (1992) A population-based study of neuroblastoma incidence, survival and mortality in North America. J Clin Oncol 10: 323–329 1732433 10.1200/JCO.1992.10.2.323

[bib2] Bergeron C, Chastagner P, Mechinaud F, Plouvier E, Defachelles AS, Dusol F, Pautard B, Edan C, Portas M, Deville A, Plantaz D, Dubourg L, Froehlich P, Rubie H (2000) Long-term renal function and hearing toxicity of CBDCA in infants treated for localized neuroblastoma (LNB). Results of the SFOP NBL 90 study. Advances in Neurobiological Research Meeting, Philadelphia, 15–18 May 2000, PC 13

[bib3] Brodeur GM, Pritchard J, Berthold F, Carlsen NLT, Castel V, Castleberry RP, De Bernardi B, Evans AE, Favrot M, Hedborg F, Kaneko M, Kemshead J, Lampert F, Lee R, Look AT, Pearson ADJ, Philip T, Roald B, Sawada T, Seegher RC, Tschuda Y, Voute PA (1993) Revisions of the international criteria for neuroblastoma diagnosis, staging and response to treatment. J Clin Oncol 11: 1466–1477 8336186 10.1200/JCO.1993.11.8.1466

[bib4] Cox DR (1977) The Analysis of Binary Data. London: Chapman & Hall

[bib5] De Bernardi B, Conte M, Mancini A, Don Francesco A, Alvisi P, Toma P, Casale F, Montezemolo LC, Cornelli PE, Carli M, Tonini GP, Pession A, Giaretti W, Garaventa A, Marchese N, Magillo P, Nigro M, Kotitsa Z, Tamaro P, Tamburrini A, Rogers D, Bruzzi P (1995) Localized resectable neuroblastoma: results of the second study of the Italian Cooperative Group for Neuroblastoma. J Clin Oncol 13: 884–893 7707115 10.1200/JCO.1995.13.4.884

[bib6] Evans AE, Albo V, D'Angio GJ, Finklesteinjz, Leiken S, Santulli T, Weiner J, Hammond GD (1976) Factors influencing survival of children with non metastatic neuroblastoma. Cancer 38: 661–666 788890 10.1002/1097-0142(197608)38:2<661::aid-cncr2820380206>3.0.co;2-m

[bib7] Fleiss JI (1981) Statistical Methods for Rates and Proportions, 2nd ed. New York: Wiley

[bib8] Garaventa A, De Bernardi B, Pianca C, Donfrancesco A, Montezemolo LC, Di Tullio MT, Bagnulo S, Mancini A, Carli M, Pession A, Arrighini A, Di Cataldo A, Tamaro P, Iasonni V, Taccone A, Rogers D, Boni L (1993) Localized but unresectable neuroblastoma: treatment and outcome of 145 cases. J Clin Oncol 11: 1770–1779 8355044 10.1200/JCO.1993.11.9.1770

[bib9] Haase GM, Wong KY, De Lorimier AA, Sather HN, Hammond GD (1989) Improvement in survival after excision of primary tumour in Stage III neuroblastoma. J Pediatr Surg 24: 194–200 2724013 10.1016/s0022-3468(89)80248-9

[bib10] Hartmann O, Scopinaro M, Tournade MF, Sarrazin D, Lemerle J (1983) Neuroblastomes traités à l' Institut Gustave Roussy de 1975 à 1979. Cent soixante treize cas. Arch Fr Pédiatr 40: 15–21 6860066

[bib11] Hero B, Simon T, Benz-Bohm G, Scheel-Walter HG, Schilling FH, Berthold F (2002) Incidence and time frame of regression in stage 2 and stage 3 neuroblastoma. Advances in Neurobiological Research Meeting, Paris, 17–19 June 2002, OC 05

[bib12] Kushner BH, Cheung NKV, Laquaglia MP, Ambros PF, Ambros IM, Bonilla MA, Gerald WL, Ladanyi M, Gilbert F, Rosenfield NS, Yeh SDJ (1996) Survival from locally invasive or widespread neuroblastoma without cytotoxic therapy. J Clin Oncol 14: 373–381 8636746 10.1200/JCO.1996.14.2.373

[bib13] Le Tourneau JN, Bernard JL, Hendren WH, Carcassonne M (1985) Evaluation of the role of surgery in 130 patients with neuroblastoma. J Pediatr Surg 20: 244–249 4009375 10.1016/s0022-3468(85)80113-5

[bib14] Peto R, Mcpherson K, Peto J (1977) Design and analysis of clinical trials requiring prolonged observation of each patient. Br J Cancer 35: 1–39 831755 10.1038/bjc.1977.1 PMC2025310

[bib15] Plantaz D, Rubie H, Michon, Mechinaud F, Coze C, Chastagner P, Frappaz D, Gigaud M, Passagia JM, Hartmann O (1996) The treatment of neuroblastoma with intraspinal extension with chemotherapy followed by surgical removal of residual disease. A prospective study of 42 cases. Results of the NBL 90 study of the French Society of Paediatric Oncology. Cancer 78: 311–319 8674009 10.1002/(SICI)1097-0142(19960715)78:2<311::AID-CNCR19>3.0.CO;2-Z

[bib16] Rosen EM, Cassady JR, Kretschmar C, Frantz CR, Levey R, Sallan SE (1984) Influence of loco-regional lymph nodes metastases on prognosis in neuroblastoma. Med Pediatr Oncol 12: 260–264 6749057 10.1002/mpo.2950120410

[bib17] Rubie H, Hartmann O, Michon J, Frappaz D, Coze C, Chastagner P, Baranzelli MC, Plantaz D, Avet Loiseau H, Benard J, Delattre O, Favrot M, Peyroulet MC, Thyss A, Perel Y, Bergeron C, Courbon-Collet B, Vannier JP, Lemerle, Sommelet D (1997) Localized neuroblastoma: *N-Myc* gene amplification is a major prognostic factor. Results of the French NBL 90 study. J Clin Oncol 15: 1171–1182 9060561 10.1200/JCO.1997.15.3.1171

[bib18] Rubie H, Michon J, Plantaz D, Peyroulet MC, Coze C, Frappaz D, Chastagner P, Baranzelli MC, Mechinaud F, Boutard P, Lutz P, Perel Y, Leverger G, De Lumley L, Millot F, Stephan JL, Margueritte G, Hartmann O (1998) Unresectable localized neuroblasroma: improved survival after primary chemotherapy including carboplatin–etoposide. Br J Cancer 77: 2310–2317 9649151 10.1038/bjc.1998.384 PMC2150389

[bib19] Rubie H, Plantaz D, Coze C, Michon J, Frappaz D, Baranzelli MC, Chastagner P, Peyroulet MC, Hartmann O (2001) Localised and unresectable neuroblastoma in infants: excellent outcome with primary chemotherapy. Med Pediatr Oncol 36: 247–250 11464897 10.1002/1096-911X(20010101)36:1<247::AID-MPO1061>3.0.CO;2-Z

[bib20] Tsuchida Y, Honna T, Kamii Y (1989) Radical excision of primary tumour and lymph nodes in advanced neuroblastoma: comparison with intensive chemotherapy. Pediatr Surg Int 6: 567–571

[bib21] West DC, Shamberger RC, Macklis RM, Kosakewich HPW, Wayne AS, Kreissman SG, Korf BR, Lavally B, Grier HE (1993) Stage III neuroblastoma over 1 year at diagnosis: improved survival with intensive multimodality therapy including multiple alkylating agents. J Clin Oncol 11: 84–90 8418247 10.1200/JCO.1993.11.1.84

